# ET-Pfam: ensemble transfer learning for protein family prediction

**DOI:** 10.1093/bioinformatics/btag121

**Published:** 2026-03-13

**Authors:** Sofia A Duarte, Rosario Vitale, Sofia Escudero, Emilio Fenoy, Leandro A Bugnon, Diego H Milone, Georgina Stegmayer

**Affiliations:** Research Institute for Signals, Systems and Computational Intelligence, sinc(i), FICH-UNL, CONICET, Ciudad Universitaria UNL, Santa Fe 3000, Argentina; Research Institute for Signals, Systems and Computational Intelligence, sinc(i), FICH-UNL, CONICET, Ciudad Universitaria UNL, Santa Fe 3000, Argentina; Research Institute for Signals, Systems and Computational Intelligence, sinc(i), FICH-UNL, CONICET, Ciudad Universitaria UNL, Santa Fe 3000, Argentina; Research Institute for Signals, Systems and Computational Intelligence, sinc(i), FICH-UNL, CONICET, Ciudad Universitaria UNL, Santa Fe 3000, Argentina; Research Institute for Signals, Systems and Computational Intelligence, sinc(i), FICH-UNL, CONICET, Ciudad Universitaria UNL, Santa Fe 3000, Argentina; Research Institute for Signals, Systems and Computational Intelligence, sinc(i), FICH-UNL, CONICET, Ciudad Universitaria UNL, Santa Fe 3000, Argentina; Research Institute for Signals, Systems and Computational Intelligence, sinc(i), FICH-UNL, CONICET, Ciudad Universitaria UNL, Santa Fe 3000, Argentina

## Abstract

**Motivation:**

Due to the rapid growth of sequence generation, which has surpassed the expert curators ability to manually review and annotate them, the computational annotation of proteins remains a significant challenge in bioinformatics nowadays. The Pfam database contains a large collection of proteins that are annotated with domain families through profile Hidden Markov models (pHMMs). Using the aligned sequences of a curated family, one HMM is trained independently for each family, missing the opportunity of learning patterns across families, i.e. from a complete view of all the dataset. As an alternative, some deep learning (DL) models have been recently proposed, nevertheless with simple representations of the inputs and moderate improvements in performance.

**Results:**

In this work, we present ET-Pfam, a novel approach based on transfer learning and ensembles of multiple DL classifiers to predict functional families in the Pfam database. Several base DL models are first trained using learned representations from protein large language models. Then, the base models are integrated using classical ensemble strategies and novel voting approaches by learning weights for each model and for each Pfam family. Results demonstrate that the proposed ET-Pfam method can consistently diminish error rates compared to individual DL models, boosting prediction performance. Among the novel ensemble strategies presented here, the learned weights by family voting achieved the best performance, with the lowest error rate (7.00%), significantly surpassing the best individual base model error (12.91%) and competitors of the state-of-the-art.

**Availability and implementation:**

Data and source code are available at https://github.com/sinc-lab/ET-Pfam.

## Graphical Abstract

**Graphical Abstract:**
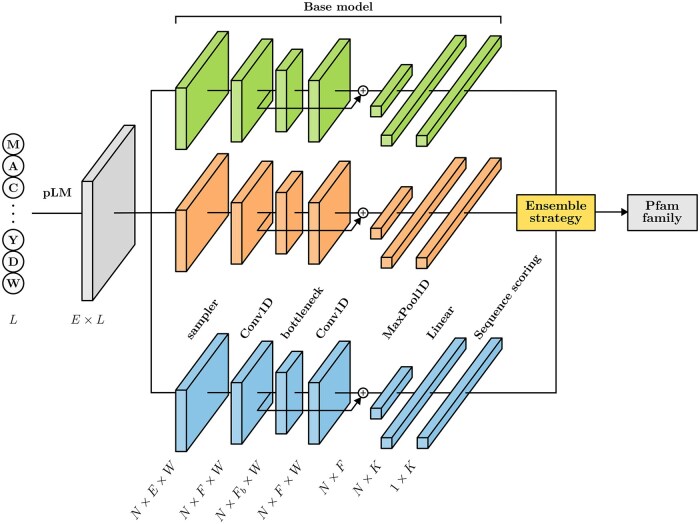

## 1 Introduction

In proteomics, the task of protein family prediction involves identifying similarity and evolutionary relationships between proteins, which is necessary for applications such as drug discovery and functional annotation of genomes (Idhaya *et al.* 2024). However, the computational complexity of the annotation task increases exponentially with the number of sequences (Wand and Jiang 1994). As of June 2025, there are 24 736 protein families and 62 646 683 sequences annotated with their corresponding family domain in the protein family database (Pfam) v37.4. However, the UniProtKB database, which constitutes the complete world of known proteins up-to-date, has 253 635 358 sequence entries. This means that it was possible to annotate the Pfam family of just ≈25% of the known protein universe.

The state-of-the-art in protein family annotation at Pfam is based on profile Hidden Markov models (pHMMs) (Eddy 1998, Bateman 2004, Eddy 2011). At Pfam, each family entry contains a seed set of representative sequences from which a pHMM is trained (Paysan-Lafosse *et al.* 2025). These pHMMs are then used to search against UniProtKB proteomes. Sequence regions that meet a family-specific curated threshold are classified with the corresponding family of the pHMM. However, in many families it is impossible to build a pHMM because the curated sequences available are just a few. Note that HMMs are single-domain generative models, meaning that each pHMM learns the underlying probability distribution from sequences of just one specific family without using information from the others, in order to generate samples that closely resemble training data for that family. Thus, such a learning approach does not take advantage of sequences from several families to learn inner patterns that might be shared among them. Moreover, profiles for training are based on manually curated sequences, and pHMM models cannot learn patterns from insufficient examples.

As a completely different approach and inspired by the recent success of deep learning (DL) models in bioinformatics (Jumper *et al.* 2021), alignment-free methods for modeling protein families with DL have appeared. DL models have the capability of learning a model from information shared across several families and can directly predict annotation from unaligned sequences. In fact, they can capture local and non-local patterns in the sequence, and transfer information across protein families. DL models are discriminative models, thus focus on learning features to define a decision boundary that separates classes within data. Instead of modeling data distribution itself, like HMMs, DL models aim to learn features with discriminative information in the training data. Thus, discriminative models are very suited for classification tasks since they can effectively capture the differences between classes. Moreover, DL models are trained looking at the complete sets of training patterns at the same time. These models are capable of inferring inner patterns or hidden rules shared across families, which can be very useful when new sequences have to be annotated. Some preliminary DL models were proposed for the Pfam annotation task (Seo *et al.* 2018, Bileschi *et al.* 2022), however relying on very simple input data representations such as one-hot encoding completed with zero padding. ProtENN (Bileschi *et al.* 2022), an ensemble of convolutional neural networks, was designed to use only a cropped segment with an isolated domain as input, i.e. it needs the beginning and ending marks of curated Pfam seeds. Both models were designed to classify one domain per sequence at a time, thus the length of the input sequence may end up being the key feature used for prediction.

On the one hand, it has been shown recently (Bugnon *et al.* 2023, Vitale *et al.* 2024) that using transfer learning from pre-trained large language models (LLMs) can reduce prediction errors in comparison to standard methods and DL models with one-hot encoding. Analogously to what happened in natural language processing, several protein language models (pLMs) have appeared in the last five years (Yang *et al.* 2018, Detlefsen *et al.* 2022, Tran *et al.* 2023) that take advantage of the vast quantity of unannotated protein sequence data available. Protein language models can capture some aspects of the grammar of the language of life as written in protein sequences (Weissenow and Rost 2025). As a matter of fact, pLMs have now turned into an important representation alternative for capturing the information encoded in a protein, thus becoming an increasingly powerful means of advancing protein prediction and annotation (Heinzinger and Rost 2025). For many applications, alignment-free pLM-based predictions now have become significantly more accurate (Weissenow and Rost 2025). Given a raw protein sequence, a pre-trained pLM can calculate a dense feature vector (embedding) that encodes its representation, which can serve as the exclusive input into downstream supervised methods greatly simplifying the modeling. Next, a predictive model can solve the downstream task by learning from embeddings associated with specific target labels. Interestingly, pLMs do not require annotations during pre-training, as they can learn from the raw protein sequences. This capability by which LLMs acquire knowledge by self-supervised learning from a large dataset (e.g. the full UniProtKB), and use it into another more specific task that requires annotated data (e.g. Pfam prediction) is named transfer learning (TL) (Weiss *et al.* 2016, Unsal *et al.* 2022). TL can be done through representation transfer, where fixed embeddings are used to feed a downstream DL model; or as a model transfer, where the pLM model is fine-tuned with the downstream task (Schmirler *et al.* 2024). Currently, one of the most used methods for fine-tuning is low-rank adaptation (LoRA) (Hu *et al.* 2022), which freezes the pre-trained model weights and introduces trainable rank decomposition matrices into each layer of the pLM, reducing the number of parameters to be adjusted.

On the other hand, the flexibility, adaptability and exceptional performance of ensemble methods together with DL have spread their application in bioinformatics research (Ganaie *et al.* 2022). In spite of these techniques being developed independently in machine learning, the recent emergence of ensemble deep learning has prompted new applications. With ensembles, instead of just building a single model, multiple and varied base models are combined to achieve better generalization performance. In the field of bioinformatics, ensembles have shown the capability to deal with small sample sizes, high dimensionality, unbalanced class distribution, and noisy and heterogeneous data generated by biological systems (Cao *et al.* 2020). However, in (Vitale *et al.* 2024) as in ProtENN (Bileschi *et al.* 2022), a classical voting schema was used for the ensemble.

In this work, we propose the combination of two strategies to improve the state-of-the art prediction of protein families in Pfam. First, to leverage the success of TL from pLMs trained in an self-supervised way from large-scale protein data. Second, to develop an ensemble of several DL models combined with TL, ET-Pfam, developing strategies beyond the classical ensemble voting, such as learned weights by model voting and learned weights by family voting, to boost prediction performance.

## 2 ET-Pfam ensemble model

**Figure 1:**
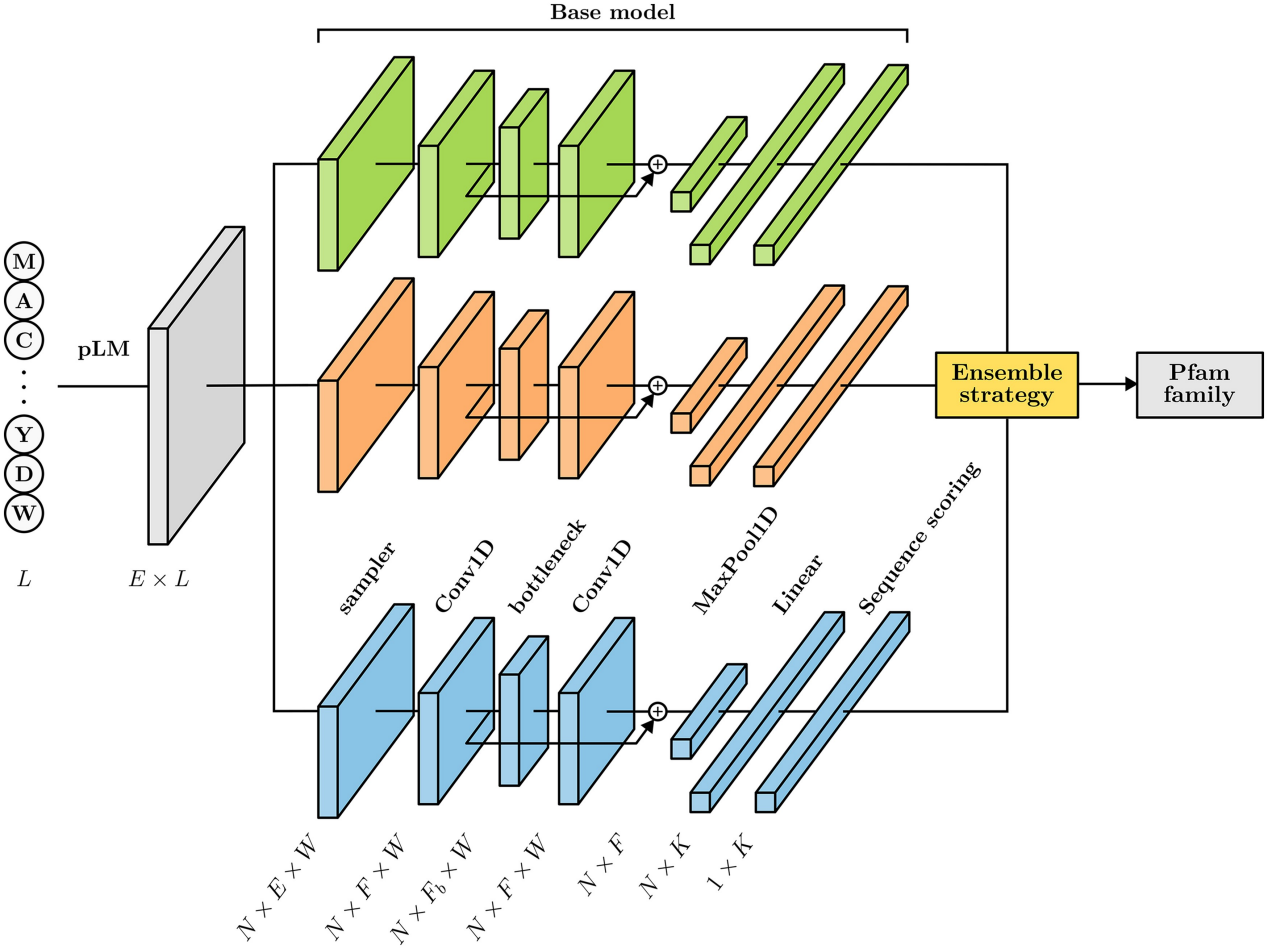
ET-Pfam architecture for Pfam family prediction in full sequences. Each input protein is represented with a sequence of embeddings, obtained from a pLM. The sequence of length *L* is sampled in smaller windows *W*, with its corresponding family class. Several base models, indicated in the figure with different colors, receive the same window embeddings and are trained separately. At the output, using the scores of base models the prediction of a Pfam family *K* can be provided according to different ensemble strategies.

The architecture of ET-Pfam is shown in Fig. 1, including the pLM, the base models of the ensemble, and the final ensemble strategy to predict the Pfam family. First, each protein sequence of length *L* in the dataset is passed through a pLM in order to obtain the corresponding embedding of size E×L, where *E* is the embedding dimension. In testing time, a window is slided with discrete steps along the protein. Each base model has a sampler that generates *N* input windows of length *W* residues from the input sequence. The base models output one family per window analyzed. When another window of the same sequence is presented to the model, it can be annotated with a different label. If for example the sequence is multi-domain, then different families can be predicted for different windows. Several base models are trained separately, indicated in the figure with different colors, having the same architecture but different hyperparameters and random initializations. In training, each window is randomly sampled. The base model has a ResNet block with a convolutional (conv1D) layer, a bottleneck, and another conv1D, with *F* filters for the first and the last convolutions, and $F_{b}$ filters in the bottleneck. After that, a max pooling along the window is performed, obtaining a vector of F×1 elements. Then there is a linear fully connected layer, with *K* output classes (the number of Pfam families), which provides the final prediction for each window. Cross-entropy loss and Adam optimizer are used during training. Each base model can be trained with its own learning rate $l_{r}$, and uses a different window length *W* in the sampler. For the final output, a Pfam family prediction can be provided according to several strategies to ensemble the output scores of the base models.

### 2.1 Base models scores

In this proposal is it important to highlight that, unlike other approaches, here the sampler extracts windows of the full protein and the convolutions are made inside each window. Given a window centered in the *n*th residue or the protein, let be $s_{ik}(n)$ the output score of the *i*th model of the ensemble, for the *k*th output class (Pfam family). For each test sequence, the simplest way of obtaining the final class score is by considering the highest score of an input window positioned at the central residue $n_{c}$. Given the beginning ($n_{b}$), ending ($n_{e}$) and the central window coordinate $n_{c}=\frac{n_{e}-n_{b}}{2}$, the central window score (CwS) is


$$s_{ik}=s_{ik}(n_{c}).$$

A better prediction can be obtained by considering the scores of the sliding windows along the test sequence. Then, the output score for each class *k* will be the maximum score along all positions *n*. That is, $s_{ik}=\max_{n}s_{ik}(n)$. However, this value can be just a single peak within the full range of the test sequence. In order to better take into account all the scores within the complete test sequence, we propose two alternatives.

The first alternative is, given the beginning ($n_{b}$) and ending ($n_{e}$) residues, to calculate the area under the $s_{i,k}(n)$ along *n*, that is the sliding window area (SwA) as


$$s_{ik}=\frac{1}{n_{e}-n_{b}}\sum_{n=n_{b}}^{n_{e}}s_{ik}(n).$$

The second alternative is to account for the coverage of the score, considering the times that each class was the winner along all the amino acids of the test sequence. The sliding window coverage (SwC) can be written as


$$s_{ik}=\frac{1}{n_{e}-n_{b}}\sum_{n=n_{b}}^{n_{e}}\delta \;\left(\arg\underset{k}{\max}s_{ik}(n),k\right),$$

where the Kroneker delta is $\delta (k^{*},k)=1$ when $k^{*}=k$ and 0 otherwise.

Finally, for each test sequence, whatever the alternative used to obtain $s_{ik}$, the predicted family $\kappa_{i}$ of the base model *i* can be obtained simply with the maximum score $\kappa_{i}=\arg\;\max_{k}\;s_{ik}$.

### 2.2 ET-Pfam ensemble voting strategies

At the output of ET-Pfam, an ensemble prediction of a determined Pfam family $\kappa^{*}$ can be provided according to different ensemble strategies.

*Simple voting:* In this voting strategy, for each possible class *k*, the sum of the base model votes is obtained and then the most voted class is selected. That is, the final predicted family $\kappa^{*}$ is


$$\kappa^{*}=\arg\underset{k}{\max}\sum_{i}\delta (\kappa_{i},k).$$

*Score voting:* Instead of considering that each base model votes with 1 or 0, the score of the output class prediction can be used to balance the voting. Therefore, the sum of the output scores of the base models is obtained, and the class with the maximum sum of scores is selected as the output prediction of the ensemble. This can be expressed simply as


$$\kappa^{*}=\arg\underset{k}{\max}\sum_{i}s_{ik}.$$

*Learned weights by model (LWM) voting:* Due to the fact that not all base models are equally good at predicting during training, in this schema the score of each base model is weighted according to


$$\kappa^{*}=\arg\underset{k}{\max}\sum_{i}w_{i}s_{ik},$$

where the weights $w_{i}$ are adjusted by gradient descent learning using the development partition. This is done after the training of the base models, using their predictions as input to the above linear model.

*Learned weights by family (LWF) voting:* As a further step along, weights of each model can be learned for each family *k* with a perceptron. In this schema, the weight $w_{ik}$ is also learned with gradient descent using the development partition. Now, the weighted output of the ensemble is


$$\kappa^{*}=\arg\underset{k}{\max}\sum_{i}w_{ik}s_{ik}+b_{k}.$$

As a variation of this strategy, for each family *k* the weighting can be learned with a more expressive model,


$$\kappa^{*}=\arg\underset{k}{\max}f_{k}(s_{k}),$$

where f(·) is a multilayer perceptron (MLP) in our experiments, and the input vector $s_{k}$ has the scores for the class *k* for all the base models.

*Stacking:* In this strategy the outputs of all the base models are concatenated to feed a family classifier. For the case of a simple perceptron the output of the ensemble is


$$\kappa^{*}=\arg\underset{k}{\max}\sum_{\ell }\sum_{i}w_{i\ell }^{k}s_{i\ell }+b_{k},$$

where each $s_{i\ell }$ is the score for the output class ℓ of the model *i* and $w^{k}$ are the weights of the kth perceptron. Similarly to the previous strategy, the outputs of all the base models can feed a more complex model, like a MLP.

## 3 Data and experimental setup

### 3.1 Data

Pfam data was used for the experiments as in (Bileschi *et al.* 2022), where seed domains of families of Pfam v.32.0 were split into train, development (dev) and test sets by clustering based on sequence identity. In order to assure remote homology (low similarity between training and testing sequences), single-linkage clustering at 25% identity within each family was used to build a clustered split. This annotation task provides a realistic scenario close to a real-world annotation problem, where testing domains might not be similar to the training ones. The clustered benchmark was chosen because all the held-out test domains are guaranteed to be far from the training set, avoiding inflated results. However, differently from ProtENN (Bileschi *et al.* 2022), in this work we have retrieved the full-length sequences. After that, we eliminated duplicate sequences present in training, development or testing sets. For example, for the family PF02228, while in the original dataset it had two seed sequences in the training set, when retrieving the full protein this family domain turned out to be inside a multi-domain protein GAG_HTL1C, which has another domain also included in the development set. Since in our rigorous validation setup the same protein cannot be part of training and development sets at the same time, that protein was eliminated from the dataset. Thus, only one protein remained for the PF02228 family in training. Additionally, due to restrictions of the pLM models used in this study, we have kept only the proteins having <1024 amino acids. This made that, e.g. for the family PF01660 only two proteins remained in the dataset. The final benchmark dataset has a total of 17 035 Pfam families and 1 096 510 full protein sequences, with 1 063 492 sequences for training, 16 675 sequences for development and 16 343 sequences for testing.

**Figure 2:**
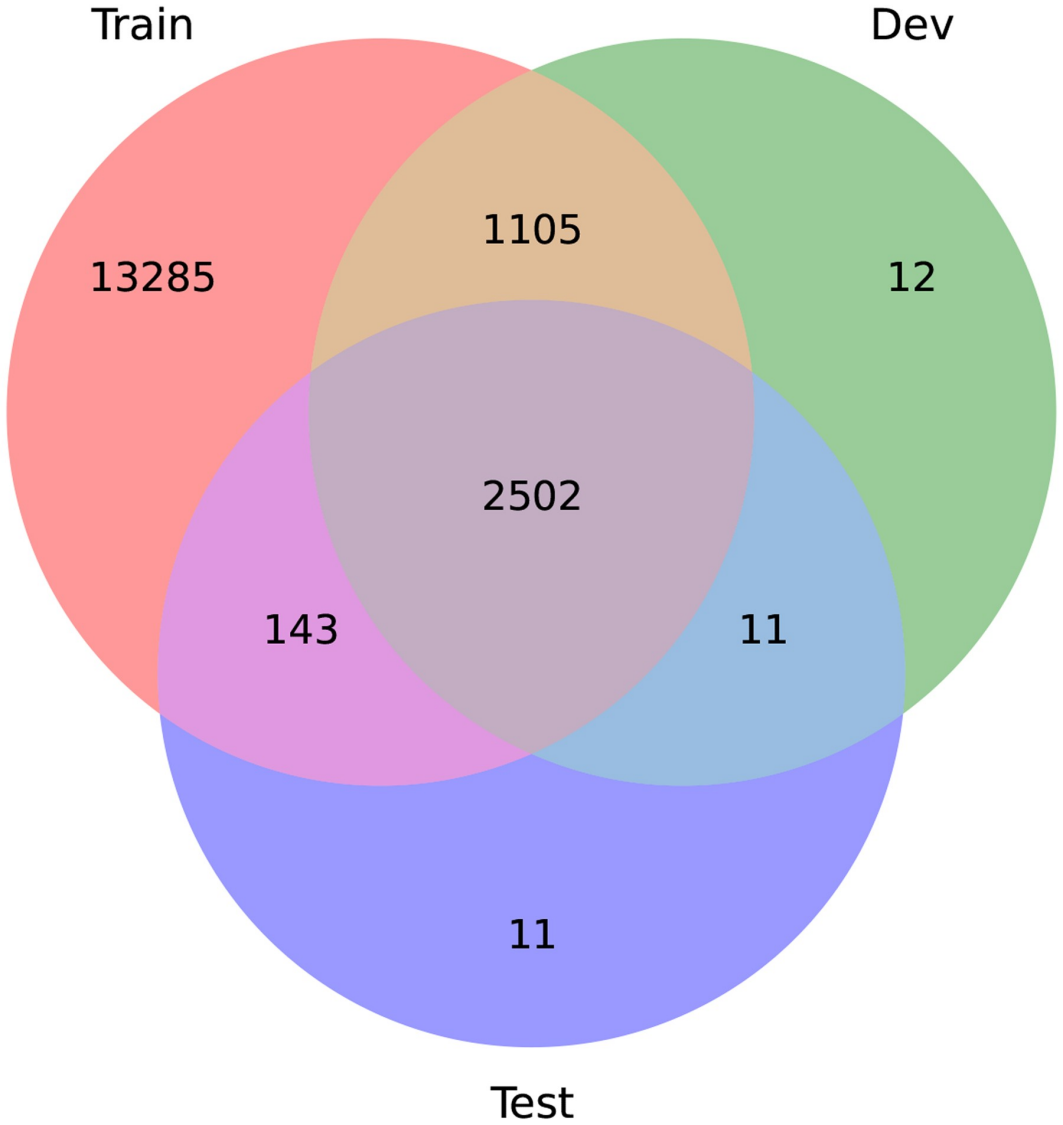
Pfam families class distribution in the full benchmark dataset (17 035 Pfam families and a total of 1 096 510 full protein sequences). The Venn diagram indicates the number of Pfam families in each partition of the dataset: training, development and testing.

This full benchmark dataset has a very large class imbalance when each possible Pfam family is considered as the output class. There are several Pfam families with just one member sequence, while there are other populated families with thousands of members. For example, the family PF13649 appears in >4000 sequences. Then, there is the family PF01660 with just 2 samples, or the family PF02228 with 1 sequence. From a total of 17 035 families, there are just 2961 families with >100 samples for training. There are 8488 families with training samples between 10 and 100; and 5586 families with <10 training samples each. Figure 2 shows a Venn diagram with the number of families at each partition (train, development and test) of the full dataset. It can be seen that, e.g. there are 11 families that are only included in the test partition, i.e. those are test families without any sequence to learn from in the training partition. There are 13 285 families with training samples that are not included in development nor test partitions. From a total of 17 035 families, only 2502 families have samples in train, development and test partition. Nevertheless, these 13 285 families only included in the train partition can be actually an advantage for the DL models, since those families provide a large variety of features and patterns to learn from. This is just opposite to the way HMMs learn, where each model learns only from samples of just one family, independently. Moreover, since there are many extra families in training, each DL model can be specialized in some types of families increasing the diversity of base models, which is well recognized as a property that improves the performance of ensembles (Pividori *et al.* 2016).

Due to the very large imbalance in the full dataset partitions, and in order to test the novel proposal first in a more controlled scenario, we have built a subset of the full benchmark dataset and named it “mini dataset.” The mini dataset families distribution was developed in order to guarantee at least 10 sequences for each family in the test partition, which lead to a total of 74 719 proteins and 395 Pfam families that are common to the train, development and test partition. The mini dataset has 56 383 sequences for train, 9076 sequences for development and 9260 sequences for test.

### 3.2 Experimental setup

We compared our proposal with the state-of-the-art prediction model currently used at Pfam for family annotation, pHMM (Eddy 2011). We also compared our proposal with a single DL model, ProtCNN, and an ensemble of DL models, ProtENN, which are both based on one-hot encoding inputs and single domains (Bileschi *et al.* 2022). As a baseline comparison, we have also included BLASTp (Altschul *et al*. 1997), one of the most well-known algorithms for similar sequences search.

To ensure a fair comparison with the HMM models, we trained pHMMs from scratch, i.e. starting from the multiple sequence alignments (MSAs) required for fitting the HMMs. We created custom MSAs using sequences from the training and development set, aligned with Muscle 3.8.31 (Edgar 2004) using its default settings. Using the MSAs generated, we then used HMMER 3.4 (Eddy 2011) to build the pHMMs for each family domain and to make the predictions over the test set. It has to be mentioned that in Bileschi *et al.* (2022) the pHMMs were built using the MSAs already provided by Pfam v32.0 including all the curated seed domains, thus those alignments included seed sequences from the complete dataset (train, dev, and test) introducing data leakage in the cross validation.

For obtaining the embeddings we followed the protocol of (Vitale *et al.* 2024). Several representation learning methods for protein sequences were experimentally benchmarked (Fenoy *et al.* 2022), and Evolutionary Scale Modeling (ESM2) (Rives *et al.* 2021, Lin *et al.* 2023) and ProtT5 (Elnaggar *et al.* 2022) proved to be the best performing ones. Therefore, esm2_t33_650M_UR50D, with size E=1,280, and prottrans_t5_xl_u50 with size *E* = 1024 were both used for obtaining embeddings. The embeddings were positionally used because, as stated in (Fenoy *et al.* 2022), the use of per-residue embeddings significantly improves the discrimination of proteins compared with averaged embeddings. Moreover, our approach makes use of pLM context coded in the per-residue embeddings to predict the protein family in each position. For pLM fine-tuning, LoRA was implemented by targeting low-rank weights to each linear layer in the ESM2 model, with a low-rank dimension of 8, scaling factor of 32 and a dropout of 0.05. This configuration produced a set of 6 M trainable parameters.

The window lengths used in the ensemble members were 32, 64, and 128, with step 4. The development partition was used for early stopping of each DL base model, with patience of 5 epochs. The learning rates explored were $l_{r}\in \{1E-4,1E-5,1E-6\}$. ResNet was configured with *F*=1100 filters for the first and the last convolutions, and $F_{b}=550$ filters in the bottleneck layer.

For each prediction model or ensemble, the error rate was calculated based on the recall (or sensitivity) as $e=1-r=1-\frac{TP}{TP+FN}$, where *TP* are the true positives, and *FN* the false negatives. We also report the $F_{1}$ score, which is the harmonic mean of precision and recall, thus summarizing both measures of performance in a single value. It is defined as $F_{1}=\frac{2TP}{2TP+FP+FN}$, where FP are the false positives.

## 4 Results

### 4.1 ET-Pfam embeddings, architectures, and ensemble strategies

As an initial assessment of the ET-Pfam in a controlled and balanced scenario, we first trained and evaluated the model and the ensemble strategies in the mini dataset, where all output classes are well represented in the three partitions: train, dev and test. The performance of base models with different hyperparameter combinations (*W* and $l_{r}$) for the test partition was predicted with centered window (CwS), sliding window area (SwA) and sliding window coverage (SwC). The best individual model with ESM2 according to CwS reached a 4.72% error, according to SwA the best individual error was 3.64%, and with SwC it was 3.66% (see Table 1, available as supplementary data at *Bioinformatics* online for details). Using ProtT5 as input embeddings, the best individual model according to CwS has 6.03% error, for SwA the best individual error was 3.98%, and with SwC it was 4.07% (details in Table 2, available as supplementary data at *Bioinformatics* online). As it can be seen, with this pLM at the input, the errors achieved are higher than with ESM2 most of the time.

**Table 1:** Ensemble strategies error at the test partition for the mini dataset when using ESM2, ESM2 fine-tuned or ESM2 and ProtT5 embeddings.^a^

	CwS			SwA			SwC		
	ESM2 (%)	ESM2 fine-tuned (%)	ESM2-ProtT5 (%)	ESM2 (%)	ESM2 fine-tuned (%)	ESM2-ProtT5 (%)	ESM2 (%)	ESM2 fine-tuned (%)	ESM2-ProtT5 (%)
Simple voting	4.40	2.37	3.83	3.79	2.81	3.37	3.79	2.81	3.37
Score voting	4.33	2.54	3.93	3.56	3.07	3.16	3.66	2.91	3.34
LWM	4.44	1.70	3.32	3.66	1.90	3.17	3.66	1.90	3.33
LWF-perceptron	**1.03**	1.24	**0.99**	1.57	1.75	1.39	1.35	1.58	1.30
LWF-MLP	1.20	1.56	1.90	1.55	4.96	2.31	1.58	3.22	2.10
Stacking Perceptron	**0.92**	1.90	**0.87**	2.54	4.54	4.14	2.56	3.54	4.90
Stacking MLP	4.78	3.78	2.96	10.54	8.31	6.29	9.89	5.43	6.13

a Best performance (lowest error) in bold.

**Table 2:** ET-Pfam ensemble strategies and competitor errors at the test partition for the full Pfam dataset (17 929 families).^a^

Input encoding	Models		**Errors** (%)		
sequence	BLASTp (Altschul *et al.* 1997)		34.94		
	pHMM (Eddy 2011)		29.28		
one-hot	ProtCNN (Bileschi *et al.* 2022)		34.21		
	ProtENN (Bileschi *et al.* 2022)		18.05		
ESM2	Single DL base model		13.94	12.91	12.98
	ET-PFam ensemble	Simple voting	12.67	11.77	11.77
		Score voting	12.34	11.56	11.45
		LWM	12.28	11.28	11.41
		LWF–perceptron	7.61	7.32	**7.00**
		LWF–MLP	8.62	9.76	8.32
ProtT5	Single DL base model		21.28	15.92	16.16
ESM2-ProtT5	ET-PFam ensemble	Simple voting	13.44	11.89	11.89
		Score voting	13.28	12.16	12.35
		LWM	12.53	11.69	11.86
		LWF–perceptron	17.30	11.80	9.59
		LWF–MLP	47.28	55.83	50.30
			**CwS**	**SwA**	**SwC**

a Best performance (lowest error) in bold.

**Figure 3:**
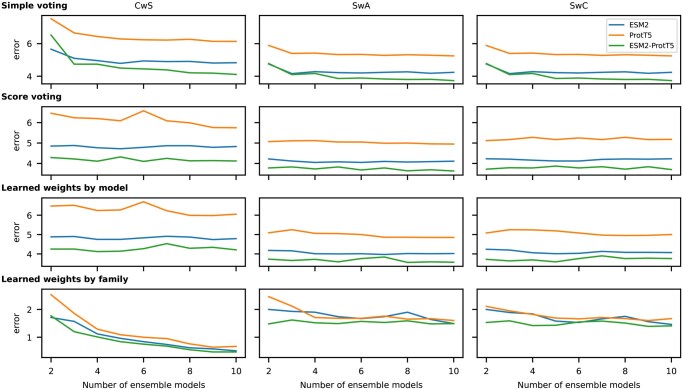
ET-Pfam classification error for different ensemble strategies (in rows) with the mini dataset development partition, using as input the ESM2 (blue), ProtT5 (orange), and ESM2 with ProtT5 (green) embeddings. In the columns: the CwS centered window score (left); SwA sliding window area (center); and SwC sliding window coverage (right).

In order to choose the best ensemble strategy for this dataset, first the errors of each ensemble strategy were calculated on the development partition, with an increasing number of members in the ensemble, up to 10 base models. Figure 3 shows the ET-Pfam ensemble error at the *y*-axis and the number of base models being incorporated at the *x*-axis. We have applied the ensemble strategies using different types of embedding as inputs to the base models: only ESM2 (blue line), only ProtT5 (orange line) and the mix of ESM2 and ProtT5, i.e. alternating models trained with a different input pLM embedding at a time (green line). The rows in this figure show: simple voting, score voting, LWM and LWF strategies, for each type of prediction window: CwS, SwA, and SwC (full results in Table 3, available as supplementary data at *Bioinformatics* online). It can be seen that the ensemble of models using only ProtT5 at the input always achieves worse results than using ESM2 only. The best results for all strategies are obtained with 10 models, with a flattened curve, and when a mix of embeddings is used in the ensemble. This is consistent with a well-known behavior in classifier ensembles, where the diversity of the members results in an overall performance that surpasses that of all the individual members. To further increase the ensemble diversity, DL base models were trained with subsampled versions of the dataset. However, since the classification results worsened in all cases, we discarded this method for further experiments.

Then, after the number of base models has been chosen using the independent development subset, the base models are ensembled with all the strategies proposed in this work and evaluated in the test partition. Results are shown in Table 1 for each strategy in rows and using ESM2 only as input embeddings for the base models, ESM2 fine-tuned with LoRA, or ESM2 and ProtT5 mixed in the ensemble in columns. When ESM2 is used as input for the base models, LWF-perceptron and Stacking perceptron, both with CwS, are the best strategies with errors 1.03 and 0.92, respectively. It can be observed that with ESM2 fine-tuned the errors achieved in the simplest voting strategies and Stacking MLP are lower than with ESM2 only, but slightly higher than ESM2 only for the best performing strategies. If models with ESM2 and ProtT5 are mixed in the ensemble, the same two strategies (LWF-perceptron and Stacking perceptron) with CwS, achieved the lowest errors.

### 4.2 ET-Pfam family prediction in the full dataset

Following the same methodology proposed in the previous subsection, we first evaluated each DL base model on the full train dataset, using the dev partition for early stopping. The errors of each ET-Pfam ensemble strategy were calculated at the development partition while base models were incrementally incorporated into the ensemble, up to 10. Members were chosen at random, only from those models with a CwS error below 20%. The selected ensemble size was 10, since at that point the performance curves flattened. Then, the DL base models were ensembled with the proposed voting strategies and evaluated in the test partition. Test results for the full Pfam dataset are discussed in this section.

Table 2 shows that when the raw sequence is used as input, BLASTp (Altschul *et al*. 1997) error is 34.94% while for pHMM (Eddy 2011) the error for the full dataset test partition is 29.28%. When one-hot input encoding is used in a single DL model such as ProtCNN (Bileschi *et al.* 2022), the prediction error is 34.21%, while the error is 18.05% when the one-hot ensemble ProtENN (Bileschi *et al.* 2022) is used. All these results are clearly improved with TL. When a single DL base model with input embeddings from ESM2 is used, the lowest SwA error achieved is 12.91% (details in Table 3, available as supplementary data at *Bioinformatics* online). Comparing this result with the error of the single model ProtCNN (one-hot based), it is evident that the gain in performance is due to the pLM embeddings used at the input. This is reinforced also by the fact that the error of a single DL base model with pLM is even lower than an ensemble of one-hot input models, the ProtENN.

Regarding ensemble strategies, it is very interesting to notice that the basic ET-Pfam ensemble voting strategies provided lower errors than the pHMM models, BLASTPp, ProtCNN, and ProtENN when using a pLM as input. Simple voting, score voting and LWM voting always have more errors than the LWF strategies. With ESM2 at the input and when the CwS is considered for predictions, the ET-Pfam LWF-perceptron error is 7.61%. When SwA is used, the error is 7.32%, and for SwC the ET-Pfam LWF-perceptron error is 7.00%, the lowest one, which represents just 1144 sequences within the complete test set of 16 343 proteins. Differently from the mini dataset, where the best was CwS, here the best is the SwC, which is more robust and has a better generalization capacity in this much larger dataset. It is interesting to analyze the reason for this additional gain in performance provided by the novel ET-Pfam ensemble strategy on top of pLMs. The per-family strategy helps to diminish the final prediction error by an impressive half because it can really leverage the knowledge of each member of the ensemble regarding each Pfam family. LWF-MLP, which was the third best method for the mini dataset, here is the second-best method after LWF-perceptron, with just over 1%–2% error. This highlights the success of LWF strategies in a more realistic validation scenario with the full dataset.

The second part of Table 2 shows the individual result of the best base model trained with ProtT5 and the mixture of ESM2 and ProtT5 embeddings, which always have higher errors than when using ESM2 only at the input. The DL base models trained only with ProtT5 embeddings were not ensembled since these ensembles always showed consistently higher errors in previous results (Fig. 3). Results of ESM2 fine-tuned on the full dataset are not provided because worse results were obtained for all cases (errors above 35%). In spite of this technique having competitive results in the mini dataset with a very controlled number of samples and classes, when working with a more realistic scenario, with large class imbalance and millions of training data samples, the pLM fine tuning overfitted the models. Stacking strategies also achieved very high errors in the full dataset. These results are not a surprise since stacking models tend to overfit to training data. This is probably due to the large number of trainable parameters (given the length of the stacked input and the 17 035 output classes), which overfitted to training data in spite of using early stopping. When the mix of embeddings are used for ensembling base models, the errors achieved are slightly higher than when using ESM2, with two exceptions: LWF-MLP provided significant higher errors (overfitting), and LWF-perceptron provided a very competitive performance in SwC (error 9.59%).

Training each base model with the full dataset, for a total of 22 epochs being ∼66 hours in total per model, took 3 hours. This process occupied, on average, 6.46 GB of RAM memory and 3.68 GB of VRAM in a NVIDIA GeForce RTX 4090. Training of the ensemble can take at most 71 min, using an average of 33 GB of RAM memory and 4 GB of VRAM (LWF-perceptron). The testing of each base model takes on average 0.0015 s per protein, while the testing of the complete ensemble for each sliding window strategy takes ∼0.015 s per protein (as expected, 10 times more because the ensemble has 10 models). This time also includes the data loading; therefore it is completely proportional to the size of the testing data. The simple voting strategy requires between 5 and 10 GB of RAM memory on average, while LWM and LWF-perceptron can take between 5 and 12 GB of RAM memory on average. Comparatively, ProtCNN takes ∼0.003 seconds per protein and uses 4.21 GB of RAM and 22.2 GB of VRAM. Since ProtENN uses 19 base models in the ensemble, it requires a total of ∼0.057 s per protein. In any case, it is important to highlight that all these models are computationally inexpensive and can be used on any computer with a commercial-grade GPU.

In summary, the proposed method has low computational requirements and comparative analysis with the state-of-the-art procedure currently used at Pfam for protein annotations has shown that ET-Pfam with the per-family strategy can provide effectively improved results, reducing the prediction error by an impressive four times in comparison to the pHMM and BLASTp techniques, and by more than half in comparison to the best DL competitor.

### 4.3 A detailed analysis of the models errors

The meaning of the reduction in prediction error from 18.05% (ProtENN) to 7.00% (ET-Pfam), and from 12.91% (best DL base model) to 7.00% (ET-Pfam) can be analyzed from a practical point of view. With ProtENN there are 376 Pfam families with 100% error, and for 214 of them the ET-Pfam model prediction is perfect (0% error). For all the proteins in these 214 Pfam families the prediction has improved from 100% to 0% error. Similarly, when a single DL base model that has a 12.91% prediction error is used, there are 413 Pfam families with 100% error. However, for 156 of those 413 families, the ET-Pfam model prediction is 0% error (see Table 5, available as supplementary data at *Bioinformatics* online and Fig. 1, available as supplementary data at *Bioinformatics* online). The detailed distributions of errors for each model can be found in Fig. 2, available as supplementary data at *Bioinformatics* online. There, it can be seen that while the DL base model has a large number of cases with 100% error, ProtENN has a larger number of errors between 10% and 90%, similarly to HMM. Thus, those models have a larger global error.

A deeper analysis into these model errors shows that even when there are highly populated families in training, the base model cannot correctly predict the test family, while ET-Pfam can and without errors. This also happens with families that have just a few training samples. More details on this can be found in Table 6, available as supplementary data at *Bioinformatics* online. Additionally, a functional analysis of the predictions is presented in Table 7, available as supplementary data at *Bioinformatics* online. This table provides details for Pfam testing families that are correctly predicted by the ET-Pfam model with the per-family strategy but are completely mistaken by the best DL base model. In these examples, it can be seen that the per-family ensemble strategy allows solving the base model confusions, which are often due to proteins that are very similar in sequence but have very distinct functions. For example, Alanine racemase N-terminal domain (PF01168) is confused by the base model with Pyridoxal-dependent decarboxylase C-terminal sheet domain (PF00278). These enzymes are homologous but catalyze different chemical reactions. They have a very similar structural conformation, and both require the same cofactor to perform their function. The N-terminal domain of both proteins is very similar (they belong to the same clan), and the same is true for the C-terminal domain. In spite of their high similarity in sequence and structure, these enzymes perform a very different function, which is not recognized by a single base model but it is indeed resolved thanks to the ET-Pfam ensemble. That is, even when considering the best of the base models (with the lowest global error), it may not have been specialized enough to distinguish between these two families. However, other models in the ensemble did it, indeed, and during the per-family weights training ET-Pfam learned which base models to prioritize in order to predict the correct family. Finally, a detail list of the 33 Pfam families that have 100% error in BLASTp, pHMM and ProtENN models, but now have 0.00% error with the ET-Pfam LWF-perceptron model, can be found in Table 8, available as supplementary data at *Bioinformatics* online. Regarding the prediction of multi-domain proteins (there are 60 in our dataset), the average error for these types of proteins is 0.24 for single DL base model, and 0.15 for ET-Pfam LWF-perceptron (details in Table 9, available as supplementary data at *Bioinformatics* online).

**Figure 4:**
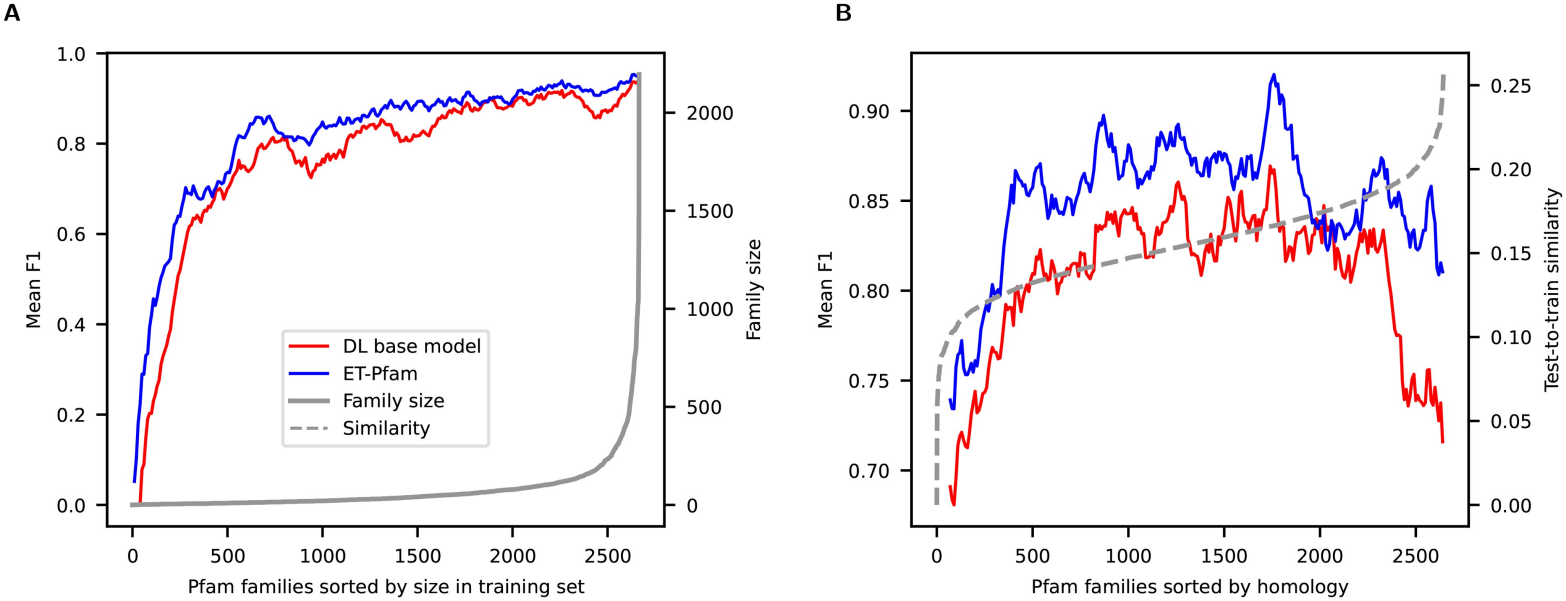
Models performance with mean $F_{1}$ score variation according to Pfam family characteristics. ET-Pfam (blue) and DL base model (red). (A) Ordered, from small (left) to large (right), according to Pfam training families size (gray curve). (B) Ordered according to test-to-train similarity (dashed gray curve).

ET-Pfam performance variation was also analyzed according to different aspects of the Pfam families. Figure 4A shows the ET-Pfam mean $F_{1}$ (blue) and the base model mean error (red) for the full dataset, ordered from small (left) to large (right) Pfam families size in training (gray curve). This figure shows that for the smallest families (those having just a few training samples, like 1 or 2) at the left of the figure, both models have a low $F_{1}$ score, but ET-Pfam has a slightly better performance than the base model. As the number of training samples per family increases to the right, both models increase performance, as it could be expected. However, the ET-Pfam model always has a higher $F_{1}$ score all along the curve. Figure 4B shows the mean ET-Pfam $F_{1}$ (blue) and the base model $F_{1}$ (red) ordered according to test-to-train similarity for the full dataset. This was calculated with BLAST, for all proteins in each test Pfam family. For each family, the similarities between all the test proteins to all the train proteins is calculated. Then, for each test protein in the family, the maximum similarity to all the train proteins is retained. Finally, the mean of these maximum similarities is used as the test-to-train similarity of the family. The right axis shows that the test-to-train similarity (homology) is at most 0.25 (as ensured by dataset design). There are some really hard samples with similarity 0.0 at the left of the figure. For those very-hard sequences, both models have similar low $F_{1}$ scores, however being ET-Pfam the model with larger $F_{1}$ scores in average along all the homologies range.

## 5 Conclusions

This work has presented ET-Pfam, a novel approach for Pfam family prediction based on an ensemble of DL base models that use transfer learning. A novel ensemble strategy learns weights for voting with the per-family output scores of DL models. Comparative results versus the classical HMM models used by Pfam and individual DL models highlighted the effectiveness of the proposed ensemble method in improving protein sequence classification, reducing the prediction error four times. Incorporating family-specific weights at the ensemble allowed the base models to better contribute to the Pfam classification. This could not have been possible with a single base model, or with the simple and classical voting combination of base models.

Results achieved have demonstrated the practical utility of embeddings from protein large language models, together with ensembles of deep learning models, for the Pfam annotation problem. We presented here a substantial advance over previous efforts applying deep learning in terms of improving the state of the art prediction and the rigorous comparison with existing methods. The next step is to use the ET-Pfam model presented here to rapidly and efficiently annotate unlabeled sequences with the aim of further expanding the annotated protein universe, which could be of special interest for biotechnology and health applications. Future work will also focus on investigating the output scores for automatic segmentation of domains.

## Supplementary Material

btag121_Supplementary_Data

## Data Availability

Data and source code are available at https://github.com/sinc-lab/ET-Pfam.
