# Supplementary material for: ET-Pfam: ensemble transfer learning for protein family prediction
Source: Bioinformatics. 2026 Mar 13;42(4):btag121. doi: 10.1093/bioinformatics/btag121 (PMC13071154; doi:10.1093/bioinformatics/btag121)
Supplement: btag121_Supplementary_Data [file btag121_supplementary_data.pdf]

ET-Pfam: Ensemble transfer learning for protein family prediction

S. A. Duarte, R. Vitale, S. Escudero, E. Fenoy, L.A. Bugnon, D.H. Milone, G. Stegmayer

Research Institute for Signals, Systems and Computational Intelligence, sinc(i), FICH-UNL, CONICET, Ciudad Universitaria UNL, 3000 Santa Fe, Argentina.

Supplementary Table 1. Individual base models error according to several possible base model score calculation criteria at the test partition for the mini dataset with input embeddings from the pLM ESM2. Best model, with lowest error, in bold.

Model	W	lr	CwS	SwA	SwC
1	32	1.00E-04	5.66%	3.73%	3.92%
2	32	1.00E-05	5.51%	3.90%	4.02%
3	32	1.00E-06	6.13%	4.12%	4.33%
4	32	1.00E-06	5.48%	**3.64%**	**3.66%**
5	32	1.00E-06	5.68%	3.99%	3.98%
6	32	1.00E-06	5.65%	3.85%	3.90%
7	64	1.00E-06	5.27%	3.65%	3.74%
8	64	1.00E-06	5.15%	3.88%	3.87%
9	128	1.00E-06	**4.72%**	4.22%	4.40%
10	128	1.00E-06	4.88%	4.05%	4.21%

Supplementary Table 2. Individual base models error according to several possible base model score calculation criteria at the test partition for the mini dataset with input embeddings from the pLM ProtT5. Best model, with lowest error, in bold.

Model	W	lr	CwS	SwA	SwC
1	32	1.00E-06	6.04%	4.67%	4.53%
2	32	1.00E-06	7.19%	4.91%	4.92%
3	32	1.00E-06	7.51%	4.74%	5.01%
4	32	1.00E-06	7.23%	4.44%	4.54%
5	32	1.00E-06	6.20%	**3.98%**	**4.07%**
6	32	1.00E-06	6.20%	4.44%	4.45%
7	32	1.00E-06	**6.03%**	4.23%	4.27%
8	32	1.00E-06	7.23%	4.60%	4.78%
9	32	1.00E-06	7.08%	4.97%	4.93%
10	32	1.00E-06	7.49%	4.57%	4.71%

Supplementary Table 3. Ensemble strategies error at the development partition for the mini dataset as each base model is being incorporated into the ensemble.

	**CwS - center window**									
**Simple voting**	**ESM2**	5.66	5.10	4.96	4.79	4.94	4.90	4.91	4.81	4.83
	**ProtT5**	7.53	6.66	6.44	6.29	6.24	6.22	6.27	6.14	6.14
	**ESM2&ProtT5**	6.53	4.74	4.74	4.50	4.45	4.39	4.21	4.19	4.11
**Score voting**	**ESM2**	4.85	4.88	4.77	4.72	4.79	4.87	4.87	4.79	4.83
	**ProtT5**	6.46	6.24	6.20	6.09	6.58	6.09	5.99	5.76	5.75
	**ESM2&ProtT5**	4.29	4.22	4.11	4.32	4.10	4.25	4.13	4.14	4.12
**LWM**	**ESM2**	4.88	4.90	4.75	4.75	4.83	4.91	4.87	4.74	4.78
	**ProtT5**	6-47	6.1	6.24	6.27	6.69	6.23	5.99	5.98	6.05
	**ESM2&ProtT5**	4.25	4.25	4.12	4.14	4.27	4.53	4.29	4.34	4.21
**LWF perceptron**	**ESM2**	1.71	1.57	1.12	0.96	0.84	0.74	0.62	0.58	0.51
	**ProtT5**	2.53	1.86	1.29	1-09	1.00	0.95	0.76	0.64	0.67
	**ESM2&ProtT5**	1.77	1.20	1.01	0.84	0.75	0.68	0.55	0.47	0.47
**LWF MLP**	**ESM2**	0.00	0.00	0.00	0.00	0.00	0.00	0.67	0.00	0.08
	**ProtT5**	60.37	10.21	0.13	0.00	31.56	29.36	86.78	82.05	27.59
	**ESM2&ProtT5**	0.00	0.00	0.00	0.00	0.00	0.00	2.11	0.31	0.19
**Stacking perceptron**	**ESM2**	0.00	0.00	0.00	0.00	0.00	0.00	0.00	0.00	0.00
	**ProtT5**	0.00	0.00	0.00	0.00	0.00	0.00	0.00	0.00	0.00
	**ESM2&ProtT5**	0.00	0.00	0.00	0.00	0.00	0.00	0.00	0.00	0.00
**Stacking MLP**	**ESM2**	0.00	0.00	0.00	0.00	0.00	0.00	0.67	0.00	0.00
	**ProtT5**	60.37	10.21	0.13	0.00	31.56	29.36	86.78	82.05	27.59
	**ESM2&ProtT5**	0.00	0.00	0.00	0.00	0.00	0.00	2.11	0.31	0.19
**Base models in the ET-PFam ensemble**		**2**	**3**	**4**	**5**	**6**	**7**	**8**	**9**	**10**

	**SwA - sliding window area**									
**Simple voting**	**ESM2**	4.75	4.16	4.28	4.22	4.20	4.24	4.27	4.18	4.24
	**ProtT5**	5.89	5.40	5.42	5.33	5.34	5.28	5.32	5.29	5.25
	**ESM2&ProtT5**	4.78	4.10	4.17	3.86	3.89	3.83	3.80	3.81	3.73
**Score voting**	**ESM2**	4.22	4.12	4.05	4.08	4.05	4.10	4.07	4.09	4.11
	**ProtT5**	5.07	5.11	5.12	5.05	5.05	4.99	5.00	4.96	4.95
	**ESM2&ProtT5**	3.78	3.83	3.73	3.83	3.68	3.78	3.64	3.69	3.63
**LWM**	**ESM2**	4.18	4.16	4.01	4.00	4.01	3.97	4.02	4.01	4.02
	**ProtT5**	5.09	5.25	5.06	5.05	5.00	4.86	4.86	4.85	4.85
	**ESM2&ProtT5**	3.73	3.66	3.72	3.59	3.76	3.84	3.56	3.59	3.57
**LWF perceptron**	**ESM2**	2.00	1.93	1.90	1.74	1.67	1.74	1.90	1.64	1.49
	**ProtT5**	2.46	2.12	1.71	1.68	1.68	1.76	1.65	1.67	1.60
	**ESM2&ProtT5**	1.48	1.62	1.52	1.49	1.57	1.53	1.59	1.48	1-49
**LWF MLP**	**ESM2**	2.20	1.93	1.80	1.74	1.69	1.52	1.47	1.69	1.68
	**ProtT5**	2.29	2.85	1.89	1.97	1.81	2.07	1.82	1.88	1.76
	**ESM2&ProtT5**	1.89	1.73	1.65	1.78	1.54	2.13	1.59	1.56	1.76
**Stacking perceptron**	**ESM2**	2.32	2.40	4.10	3.98	4.27	3.17	2.52	2.21	2.29
	**ProtT5**	2.85	3.63	3.58	2.18	6.49	3.32	3.35	2.80	3.30
	**ESM2&ProtT5**	2.99	2.67	2.90	2.22	1.91	1.73	2.21	2.29	2.50
**Stacking MLP**	**ESM2**	2.11	2.44	3.87	3.94	3.97	8.05	6.30	7.57	10.38
	**ProtT5**	68.4	27.89	5.23	3.77	45.95	49.37	88.73	85.85	47.2
	**ESM2&ProtT5**	1.87	1.81	1.75	3.68	3.81	4.46	7.44	5.73	5.93
**Base models in the ET-PFam ensemble**		**2**	**3**	**4**	**5**	**6**	**7**	**8**	**9**	**10**

	SwC - sliding window coverage									
**Simple voting**	**ESM2**	4.75	4.16	4.28	4.22	4.20	4.24	4.27	4.18	4.24
	**ProtT5**	5.89	5.40	5.42	5.33	5.34	5.28	5.32	5.29	5.25
	**ESM2&ProtT5**	4.78	4.10	4.17	3.86	3.89	3.83	3.80	3.81	3.73
**Score voting**	**ESM2**	4.23	4.21	4.16	4.12	4.12	4.20	4.22	4.21	4.23
	**ProtT5**	5.12	5.17	5.28	5.17	5.25	5.17	5.28	5.17	5.18
	**ESM2&ProtT5**	3.72	3.79	3.78	3.87	3.78	3.84	3.72	3.84	3.70
**LWM**	**ESM2**	4.24	4.20	4.06	4.01	4.03	4.13	4.08	4.08	4.07
	**ProtT5**	5.08	5.25	5.24	5.19	5.08	4.97	4.95	4.96	5.00
	**ESM2&ProtT5**	3.72	3.64	3.69	3.59	3.76	3.90	3.76	3.78	3.76
**LWF perceptron**	**ESM2**	2.00	1.89	1.84	1.58	1.53	1.66	1.75	1.56	1.46
	**ProtT5**	2.11	1.95	1.82	1.69	1.66	1.71	1.67	1.60	1.67
	**ESM2&ProtT5**	1.53	1.59	1.42	1.43	1.55	1.58	1.51	1.39	1.41
**LWF MLP**	**ESM2**	2.19	1.89	1.78	1.74	1.58	1.57	1.43	1.62	1.51
	**ProtT5**	2.06	2.02	1.82	1.77	1.80	1.96	1.66	1.87	1.55
	**ESM2&ProtT5**	1.44	1.57	1.46	1.47	1.58	1.70	1.56	1.57	1.65
**Stacking perceptron**	**ESM2**	3.29	2.55	4.57	4.58	4.95	3.67	3.10	2.42	2.91
	**ProtT5**	3.55	4.72	4.30	2.86	6.55	3.51	3.57	3.12	3.77
	**ESM2&ProtT5**	4.05	3.22	3.22	2.66	2.92	2.04	2.79	2.64	2.91
**Stacking MLP**	**ESM2**	2.35	2.84	4.23	4.09	3.78	6.97	6.10	7.62	10.01
	**ProtT5**	68.39	25.18	5.18	3.10	44.41	43.95	89.01	83.74	43.71
	**ESM2&ProtT5**	2.11	2.15	1.88	3.64	3.88	4.32	7.56	5.10	5.28
**Base models in the ET-PFam ensemble □**		**2**	**3**	**4**	**5**	**6**	**7**	**8**	**9**	**10**

Supplementary Table 4. Individual models error according to several possible base model score calculation criteria at the test partition for the full dataset with input embeddings from the pLM ESM2. Best model, with lowest error, in bold. The models used in the final ensemble are indicated with *.

Model	W	lr	CwS	SwA	SwC
1	32	1.00E-04	25.87%	20.86%	21.34%
2		1.00E-05	22.76%	18.18%	18.49%
3		1.00E-06	27.00%	20.56%	21.18%
4	64	1.00E-04	21.95%	18.67%	18.81%
5		2.00E-04	18.94%	15.77%	16.02%
6		3.00E-04	26.64%	21.30%	21.72%
7		1.00E-05	20.94%	17.00%	17.42%
8		2.00E-05	21.59%	18.26%	18.45%
9		3.00E-05	21.56%	18.29%	18.11%
10		1.00E-06	21.23%	16.74%	17.24%
11	128	1.00E-04	18.46%	16.17%	16.31%
12		2.00E-04	21.05%	18.26%	18.55%
13*		3.00E-04	16.17%	14.20%	14.22%
14*		3.00E-04	15.74%	13.75%	13.91%
15*		1.00E-04	13.94%	**12.91%**	12.98%
16*		1.00E-04	14.73%	13.30%	13.07%
17*		1.00E-04	15.00%	13.03%	12.99%
18*		1.00E-04	14.72%	13.56%	13.43%
19*		1.00E-05	15.44%	14.04%	14.28%
20*		1.00E-05	15.36%	13.88%	13.74%
21*		1.00E-05	15.64%	13.97%	14.08%
22*		1.00E-05	14.94%	13.69%	13.58%
23		1.00E-05	16.79%	14.77%	15.11%
24		1.00E-05	16.94%	15.24%	15.50%
25		2.00E-05	16.89%	14.97%	15.56%
26		3.00E-05	17.30%	15.64%	15.89%
27		1.00E-06	20.31%	16.84%	17.45%

Supplementary Table 5. Number of Pfam families with 100% error in each competitor method, where ET-Pfam has 0% error.

Method	Families with 100% error	Families recovered with ET-Pfam
BLASTp	627	371
HMM	397	215
ProtENN	376	214

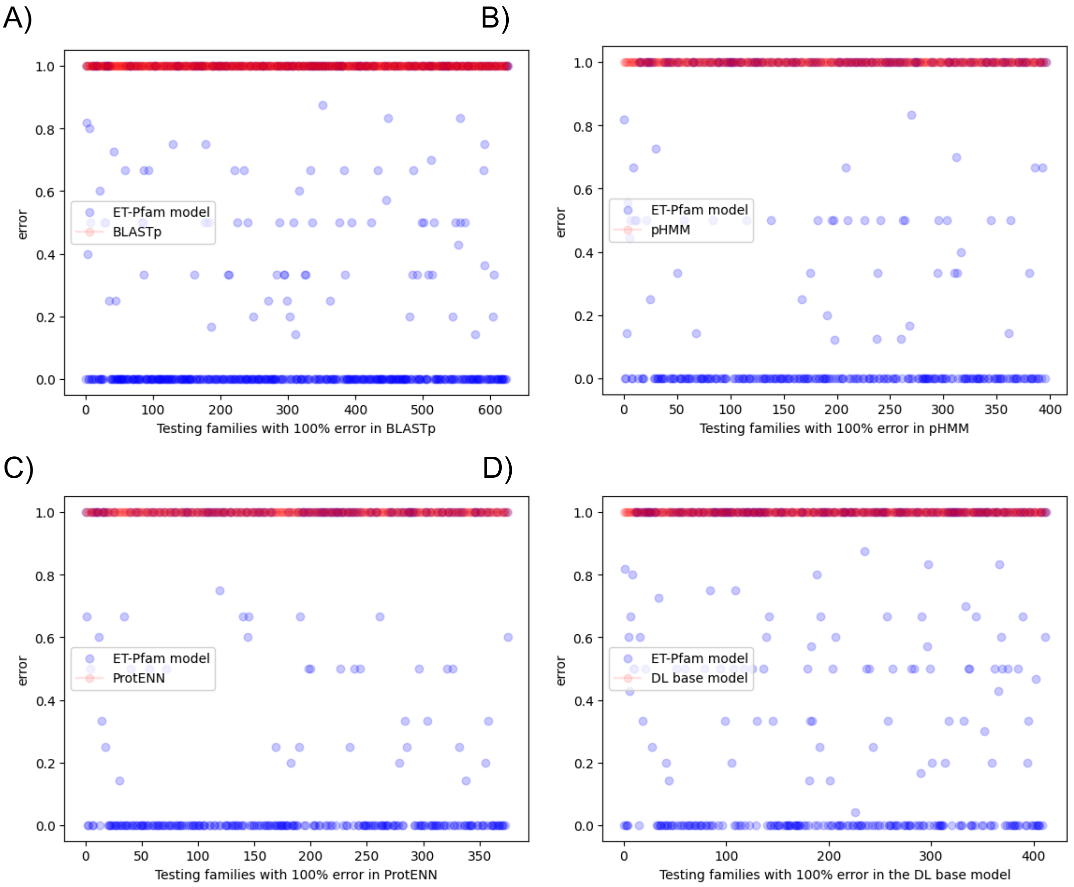

Supplementary Figure 1. Detailed error rate of each competitor method (red) and ET-Pfam LWF perceptron model (blue) for testing Pfam families with error=1.0 at the competitor method. A) BLASTp. B) pHMM. C) ProtENN. D) DL base model.

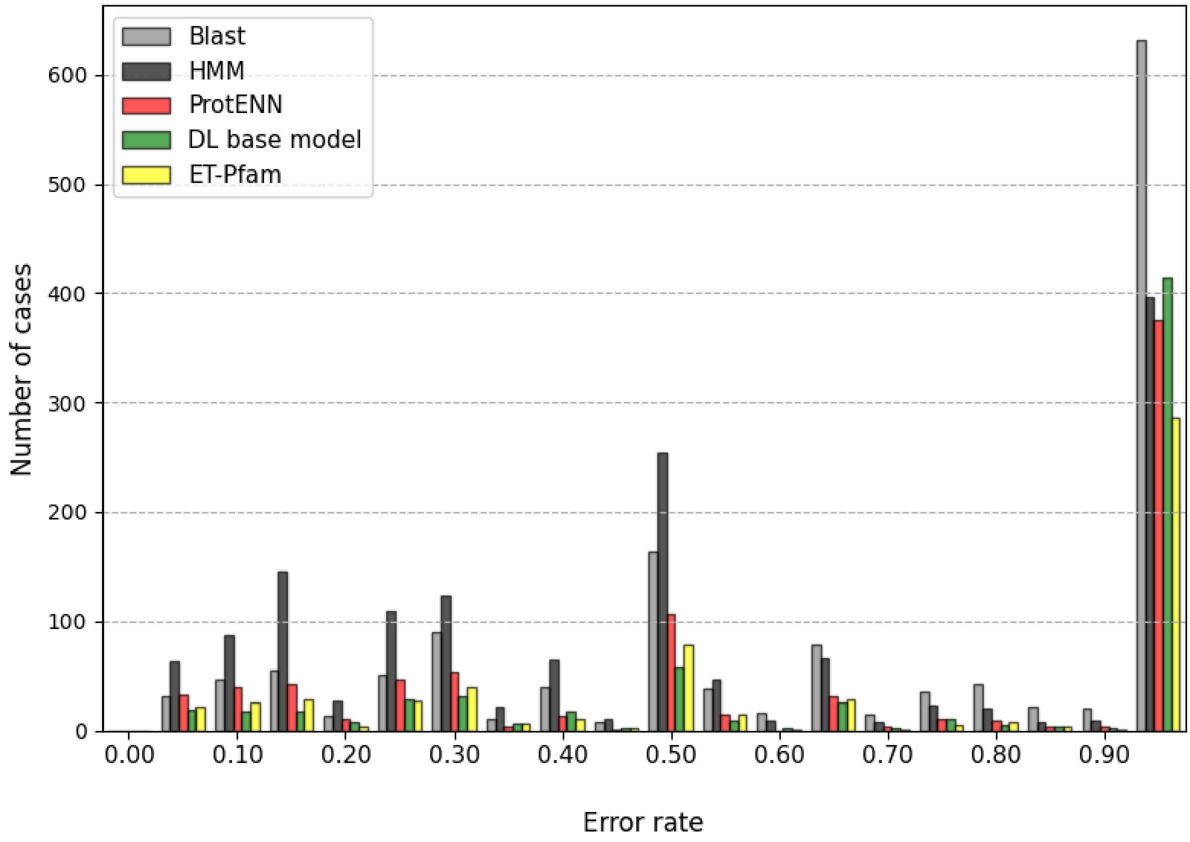

Supplementary Figure 2. Error distributions per model for the full Pfam test set.

Supplementary Table 6. Details of some Pfam families that have 100% or similar error in the best DL base model but 0% or very few errors in the ET-Pfam model with LWF perceptron. For each PFam family it is shown the family name, a brief description of its function, the number of corresponding training and testing sequences in the data set, and the corresponding prediction error for the best DL base model and the ET-Pfam with LWF perceptron model. The rows are ordered in descending order according to the training data seized for each Pfam family. The ET-PFam with the per-family voting strategy can improve results for those families, even with fewer training samples, since it combines the best scores from the best individual models for each family.

			Data samples		Error	
**Pfam family**	**Family name**	**Description**	**Train**	**Test**	**Base model**	**ET-Pfam**
**PF01168**	Alanine racemase, N-terminal domain	This family is important for bacterial cell wall biosynthesis, as it catalyzes the reversible conversion between L-alanine and D-alanine isomers.	273	11	0.546	0.000
**PF01416**	tRNA pseudouridine synthase	This family includes enzymes responsible for the conversion of uridine to pseudouridine in tRNA molecules. This stabilizes local tRNA structure, enhances proper tRNA-ribosome interactions and contributes to translational accuracy and efficiency.	251	21	0.810	0.000
**PF14450**	Cell division protein FtsA	It is essential for bacterial cell division, and co-localises to the septal ring with FtsZ.	233	13	1.000	0.000
**PF13012**	Maintenance of mitochondrial structure and function	This is C-terminal to the Mov24 region of the yeast proteasomal subunit Rpn11 and seems likely to regulate the mitochondrial fission and tubulation processes, i.e. the outer mitochondrial membrane proteins.	230	33	1.000	0.000
**PF13233**	Complex1_LYR-like	This is a family of proteins carrying the LYR motif of family Complex1_LYR, likely to be involved in Fe-S cluster biogenesis in mitochondria.	196	8	1.000	0.000
**PF02498**	BRO family, N-terminal domain	This family includes the N-terminus of baculovirus BRO and ALI motif proteins. It has been suggested that BRO are DNA binding proteins that influence host DNA replication and/or transcription.	149	9	1.000	0.000
**PF07687**	Peptidase dimerisation domain	This domain consists of 4 beta strands and two alpha helices which make up the dimerisation surface of members of the M20 family of peptidases, which are Glutamate carboxypeptidases, a bacterial enzyme with applications in cancer therapy.	133	14	1.000	0.000
**PF03061**	Thioesterase superfamily	A wide variety of enzymes contain this domain, principally thioesterases. Proteins containing this domain include 4HBT which catalyses the final step in the soil dwelling microbe Pseudomonas CBS-3.	93	19	1.000	0.000

**PF08489**	TiaS FLD domain	This is the FLD domain found in an archaeal ATP-dependent agmatine transferase that catalyses the formation of agm2C at the wobble position of tRNA. This modified base specifically recognises AUA codons.	92	14	1.000	0.000
**PF08647**	BRE1 E3 ubiquitin ligase	It is directly involved in chromatin regulation via histone ubiquitination, serving as a signal to open up the chromatin structure, allowing other enzymes to access the DNA, directly regulating gene transcription, DNA repair and epigenetic regulation.	90	15	1.000	0.000
**PF12706**	Beta-lactamase superfamily domain	Beta-lactamases are enzymes that inactivate beta-lactam antibiotics. Metallo beta lactamases exhibit low sequence identity between enzymes but they are structurally similar. They have a characteristic α-β/β-α sandwich fold in which the active site is at the interface between domains.	69	24	1.000	0.000
**PF00061**	Lipocalin / cytosolic fatty-acid binding protein family	Lipocalins are a family of proteins that bind and transport small hydrophobic molecules, such as lipids, steroid hormones, bilins, and retinoids. Found across species, they play a relevant role in metabolism and signaling.	57	11	1.000	0.000
**PF01087**	Galactose-1-phosphate uridyl transferase, N-terminal domain	This is an enzyme responsible for converting ingested galactose to glucose. It catalyses the conversion of UDP-glucose during galactose metabolism. Defects in this enzyme in humans is the cause of galactosemia, an inherited disorder of galactose metabolism that leads to jaundice, cataracts and mental retardation.	49	13	1.000	0.000
**PF00091**	Tubulin/FtsZ family, GTPase domain	This family is fundamental to cell division and intracellular organization. These proteins can self-assemble into long polymers. In eukaryotes, they manage intracellular transport. In bacteria, FtsZ forms a structure that acts as the essential machinery for self division.	44	13	0.923	0.000
**PF00092**	von Willebrand factor type A domain	This protein-binding domain mediates protein-protein interactions, and is involved in adhesion, signaling, transport, transcription and DNA repair. Mutations in this domain are associated with several human diseases.	32	15	1.000	0.467

Supplementary Table 7. Analysis, from a functional point of view, of some Pfam testing families that are all correctly predicted by the ET-Pfam model with per-family strategy, but are completely mistaken by the best DL base model. For each Pfam family in the test, its name and the incorrect family predicted by the base model is presented. The last column presents an analysis of their functional differences. In these selected examples, it can be seen that the per-family ensemble strategy allows solving the base model confusions, which are often due to proteins that are very similar in sequence, but however have very distinct functions.

Pfam family in test	Name	Pfam family predicted	Name	Description
**PF01416**	tRNA pseudouridine synthase	**PF10105**	Uncharacterized protein conserved in bacteria (DUF2344)	PF10105 and PF01416 both belong to the PseudoU_synth clan (CL0649). PF01416 corresponds to functional tRNA pseudouridine synthases.<br><br>However, PF10105 is distantly related to that family, and lacks the conserved catalytic aspartame residue, suggesting a non-catalytic or auxiliary role associated with RNA-related processes.
**PF00091**	Tubulin/FtsZ family, GTPase domain	**PF10644**	Misato Segment II tubulin-like domain	The family Tubulin/FtsZ (PF00091) is fundamental to cell division and intracellular organization. In eukaryotes, tubulin is the major component of microtubules, which manage the transport of organelles and vesicles, guide chromosome separation and provide cell shape and motility. In bacteria, FtsZ forms the cytokinetic ring that acts as the essential machinery for self division.<br><br>Differently, the Misato protein (PF10644) is involved in the regulation of mitochondrial distribution and morphology. It contains three domains. Segment II aligns with myosin heavy chain sequences. Myosins are a family of ATP-dependent motor proteins that convert energy into mechanical force to drive cellular movement and transport.
**PF08647**	BRE1 E3 ubiquitin ligase	**PF13870**	CCDC113/CCDC96, coiled-coil	The BRE1 E3 ubiquitin ligase (PF08647) is involved in the regulation of chromatin, the packaged structure of the genome inside the nucleus, via histone ubiquitination. This modification acts as a signal to open up the chromatin structure, allowing other enzymes to access the DNA, directly regulating gene transcription, DNA repair and epigenetic regulation.<br><br>The family CCDC113/CCDC96 (PF13870) operates in a completely different part of the cell, the cilia and flagella, which are structures that project from the cell surface. In this context, they play a structural role in controlling cellular motility.<br><br>The most apparent link between these two families is that they both contain regions enriched in coiled-coil structures. This similarity is purely structural, as their biological roles are different.
**PF14450**	Cell division protein FtsA	**PF02491**	SHS2 domain inserted in FtsA	FtsA (PF14450) plays a central role in bacterial cell division. It acts as a mechanical anchor to the membrane for the cytokinetic ring, a molecular structure that forms at the cell's center, and acts as a scaffold that constricts the cell membrane, allowing the separation of the bacterium into two daughter cells.

				Within the FtsA protein, there is a specific insertion that folds into a distinct globular module, which is the SHS2 domain (PF02491). While FtsA provides the overall structural anchor and energy, the SHS2 domain is primarily responsible for mediating protein-protein interactions, facilitating both homo- and heteromeric contacts within the division complex.
**PF01168**	Alanine racemase, N-terminal domain	**PF00278**	Pyridoxal-dependent decarboxylase, C-terminal sheet domain	The enzyme Alanine racemase is important for bacterial cell wall biosynthesis, as it catalyzes the reversible conversion between L-alanine and D-alanine isomers. This enzyme consists of two domains, one of them being an N-terminal domain (PF01168)).<br><br>Differently, the PF00278 domain belongs to the C-terminal sheet region of pyridoxal-dependent decarboxylases. These enzymes act on substrates such as ornithine, lysine, and arginine to remove a carboxyl group.<br><br>These enzymes, Alanine racemase and pyridoxal-dependent decarboxylase, are homologous. They catalyze different chemical reactions, but have a very similar structural conformation, and both require the same cofactor to perform their function. The proteins are composed of two domains: an N-terminal domain with an α/β barrel fold, and a C-terminal domain composed of β-sheets. The N-terminal domain of both proteins is very similar (they belong to the same clan), and the same is true for the C-terminal domain.<br>In spite of their high similarity in sequence and structure, these enzymes perform a very different function, which is not recognised by a single base model but it is indeed resolved thanks to the ET-Pfam ensemble per-family strategy.

Supplementary Table 8. Top-33 Pfam families that have 100% error in BLASTP, pHMM and ProtENN models, but now have 0.00% error with the ET-Pfam LWF perceptron model.

PFam family	Description
PF14269	Arylsulfotransferase (ASST). Group of arylsulfotransferase-like proteins from eukaryotes, archaea and bacteria.
PF01513	ATP-NAD kinase N-terminal domain. Members of this family catalyses the phosphorylation of NAD to NADP utilising ATP and other nucleoside triphosphates as well as inorganic polyphosphate as a source of phosphorus.
PF06777	Helical and beta-bridge domain. This family represents a conserved region within a number of eukaryotic DNA repair helicases.
PF13420	Acetyltransferase (GNAT) domain. Class of transferase enzymes that transfers an acetyl group in a reaction called acetylation. Post-translational modification of a protein via acetylation can transform its functionality by altering various properties like hydrophobicity, solubility, and surface attributes.
PF18851	Bacterial archaeo-eukaryotic release factor family 8. Likely to play roles in biological conflicts or regulation under stress conditions at the ribosome.
PF00959	Phage lysozyme. This family includes lambda phage lysozyme and E. coli endolysin.
PF14457	Prokaryotic E2 family A. Member of the E2/UBC superfamily of proteins found in several bacteria. The protein is usually encoded in the gene neighborhood of a distinct metallobetalactamase family gene.
PF02414	Borrelia ORF-A. This protein is encoded by an open reading frame in plasmid borne DNA repeats of Borrelia species. The function of this putative protein is unknown.
PF07922	Glycosyltransferase family 52. This family includes several bacterial glycosyltransferases. A member of this family is involved in a step of lipooligosaccharide biosynthesis requiring sialic acid transfer; these lipooligosaccharides are thought to be important in the process of pathogenesis.
PF14558	ML-like domain. This domain may be involved in lipid binding.

PF12413	Homeobox protein distal-less-like N terminal. This family is the N terminal of a homeobox protein involved in embryonic development and adult neural regeneration.
PF15956	Domain of unknown function (DUF4760). This entry represents an uncharacterised alpha helical domain found in bacteria, archaea and viruses.
PF08875	Domain of unknown function (DUF1833). This family of proteins are functionally uncharacterised and are predicted to adopt an all-beta fold.
PF07524	Bromodomain associated. This domain is predicted to bind DNA and is often found associated with Bromodomains and in transcription factors.
PF14997	CECR6/TMEM121 family. This family includes a protein which has been identified in a screen for candidate genes for the developmental disorder Cat Eye Syndrome. The function of this family is unknown.
PF12605	Casein kinase 1 gamma C terminal. CK1gamma is a membrane-bound casein kinase 1 enzyme. Experiments show that this protein is both necessary and sufficient to transduce LRP6 signalling in vertebrates and *Drosophila* cell.
PF15780	Abnormal spindle-like microcephaly-associated, ASPM-SPD-2-Hydin. This domain is found in proteins associated with cilia, flagella, the centrosome and the Golgi complex. Defects in proteins containing this domain, such as Hydin and OCRL, have been linked to hydrocephalus and Lowe oculocerebrorenal syndrome (OCRL,) respectively.
PF12369	Gonadotropin hormone receptor transmembrane region. This family contains the transmembrane region of Follicular stimulating hormone and luteinizing hormone, the two major gonadotropin hormone receptors, involved in development and maturation of germ cells in both fecund genders.
PF13856	ATP-binding sugar transporter from pro-phage. Members of this family are putative ATP-binding sugar transporter-like protein.
PF18740	EC042_2821-like Restriction Endonuclease-like domain. This entry represents a Restrictions Endonuclease-like fold fused to a HEPN-like domain.
PF18540	Domain of unknown function (DUF5626). This is a domain of unknown function mostly found in firmicutes.
PF12927	Domain of unknown function (DUF3835). This is a C-terminal domain conserved in fungi.

PF01232	Mannitol dehydrogenase Rossmann domain. Mannitol 2-dehydrogenase catalyses the NAD-dependent reduction of mannitol-1-phosphate to fructose-6-phosphate as part of the phosphoenolpyruvate-dependent phosphotransferase system.
PF05072	Herpesvirus UL43 protein. UL43 genes are expressed with true-late (gamma2) kinetics and have been identified as a virion tegument component.
PF01126	Heme oxygenase. It is the enzyme that carries out the oxidation of haem, it cleaves the haem ring at the alpha-methene bridge to form biliverdin and carbon monoxide.
PF07022	Bacteriophage CI repressor helix-turn-helix domain. This family consists of several phage CI repressor proteins and related bacterial sequences.
PF03210	Paramyxovirus P/V phosphoprotein C-terminal. This family includes phosphoprotein P and the non-structural phosphoprotein V from different paramyxoviruses. The P protein is essential for the activity of the viral RNA polymerase complex.
PF07934	8-oxoguanine DNA glycosylase, N-terminal domain. This enzyme is found in archaeal, bacterial and eukaryotic species, and is specifically responsible for the process which leads to the removal of 8-oxoguanine residues.
PF14875	N-term cysteine-rich ER, FAM69. A family of transmembrane proteins that localize to the endoplasmic reticulum in cultured cells and are predicted to have a protein kinase structure and function There are currently few indications of the involvement of this family members in human diseases.
PF02973	Sialidase, N-terminal domain. These are a widespread group of enzymes that hydrolyse the glycosidic bond between two or more carbohydrates, or between a carbohydrate and a non-carbohydrate moiety.
PF18745	Secreted Novel AID/APOBEC-like Deaminase 2. A family of secreted AID/APOBEC like deaminases found in ray-finned fishes.
PF12857	TOBE-like domain. Probably involved in the recognition of small ligands such as molybdenum and sulfate. It is found in ABC transporters immediately after the ATPase domain.
PF12615	F sex factor protein N terminal. This domain family is found in bacteria, with possible DNA binding domains.

Supplementary Table 9. Detail on the errors of the best DL base model and the ET-Pfam model with LWF for the Pfam families that have multi-domain proteins (60). Proteins that are correctly predicted by both models (45) and incorrectly predicted by both models (6) are not shown. The average error for these types of proteins is 0.24 for the best single DL base model, and it is 0.15 for ET-Pfam LWF. It can be seen that the single base model has a larger number or errors in these types of proteins than the ET-Pfam model.

Multi-domain proteins	DL base model error	ET-Pfam LWF error
PF02847	1.0	0.0
PF02779	1.0	0.0
PF09240	1.0	0.0
PF00700	1.0	0.0
PF16653	1.0	0.0
PF13144	1.0	0.0
PF00561	1.0	0.0
PF11647	1.0	0.5
PF13426	0.5	0.5
PF08448	0.5	1.0
PF10554	0.5	1.0
